# Roles of P300 and Late Positive Potential in Initial Romantic Attraction

**DOI:** 10.3389/fnins.2021.718847

**Published:** 2021-10-14

**Authors:** Guangjie Yuan, Guangyuan Liu, Dongtao Wei

**Affiliations:** ^1^College of Electronic and Information Engineering, Southwest University, Chongqing, China; ^2^Chongqing Collaborative Innovation Center for Brain Science, Chongqing, China; ^3^Institute of Affective Computing and Information Processing, Southwest University, Chongqing, China; ^4^Chongqing Key Laboratory of Nonlinear Circuits and Intelligent Information Processing, Chongqing, China; ^5^Faculty of Psychology, Southwest University, Chongqing, China; ^6^Key Laboratory of Cognition and Personality, Ministry of Education, Chongqing, China

**Keywords:** romantic attraction, mate selection, late positive potential, P300, emotion processing, attention control

## Abstract

Initial romantic attraction (IRA) refers to a series of positive reactions to potential romantic partners at the initial encounter; it evolved to promote mate selection, allowing individuals to focus their mating efforts on their preferred potential mates. After decades of effort, we now have a deeper understanding of the evolutionary value and dominant factors of IRA; however, little is known regarding the brain mechanisms related to its generation and evaluation. In this study, we combined classic event-related potential analysis with dipole-source analysis to examine electroencephalogram (EEG) signals generated while participants assessed their romantic interest in potential partners. The EEG signals were categorized into IRA-engendered and unengendered conditions based on behavioral indicators. We found that the faces elicited multiple late positivities, including P300 over the occipital–parietal regions and late positive potentials (LPPs) over the anterior regions. When compared to faces that did not engender IRA, faces that did engender IRA elicited (1) enhanced P300 over the parietal regions and heightened neural activity in the insula and cingulate cortex and (2) larger LPPs over the anterior regions and heightened neural activity in the orbitofrontal cortex, dorsolateral prefrontal cortex, cingulate cortex, frontal eye field, visual cortex, and insula. These results suggest IRA is generated and evaluated by an extensive brain network involved in emotion processing, attention control, and social evaluations. Furthermore, these findings indicate that P300 and LPP may represent different cognitive processes during IRA.

## Introduction

The lyrics from “You Had Me From Hello” by American country music artist Kenny Chesney describe what many people experience the first moment they meet someone: They are deeply attracted; their eyes are fixed on this new person, and they forget everything else; inexplicable excitement and pleasure fill their body. In that moment, the seeds of love sprout quietly. As described in the lyrics, attraction to a person in initial encounter is traditionally the first step in any romantic relationship (Olderbak et al., 2017; Gerlach and Reinhard, 2018). This kind of attraction, which occurs in the context of initial or brief encounters, has inconsistently been referred to as initial romantic attraction (IRA) (Finkel et al., 2007), romantic interest at zero acquaintance (Asendorpf et al., 2011), and love at first sight (Swami, 2013; Barelds and Barelds-Dijkstra, 2016). Here the term IRA will be used to describe this attraction. IRA is one of the three main emotional motivational systems that mammals display during mating and reproduction. It is characterized by increased energy and focused attention on one or more potential mates, and in humans, it is usually accompanied by feelings of excitement, “intrusive thinking” about a partner, and a desire to emotionally bond with a potential partner (Fisher, 1998; Fisher et al., 2002).

From the perspective of evolutionary biology, IRA evolved to enable an individual to choose a specific mating partner that he/she prefers among potential partners, thereby reducing the waste of mating resources and increasing the individual’s chances of success in the highly competitive mating market (Fisher et al., 2002, 2005; Place et al., 2009). The IRA not only plays an important role in specific preferred mating partner selection, but also has a significant impact on the quality of subsequent romantic relationships (Finkel et al., 2007; Hefner and Wilson, 2013; Zsok et al., 2017). Therefore, in past decades, social psychologists have conducted a large number of fruitful behavioral studies (Luo and Zhang, 2009; Asendorpf et al., 2011; Maner and Ackerman, 2015; Olderbak et al., 2017; Gerlach and Reinhard, 2018). These studies not only showed that the generation of IRA is influenced by many factors, such as attractiveness and likability (Finkel et al., 2007; Asendorpf et al., 2011; Olderbak et al., 2017), but also indicated that these factors play important and different roles in the process of romantic attraction. For example, although consensus judgments of a potential partner’s physical attractiveness (PA) were thought to predict the potential partner’s popularity (probability of being selected by the opposite sex), individual judgment of a potential partner’s likability was thought to be the dominant factor influencing participants’ mate choice (whether to interact further with the potential partner) (Finkel et al., 2007; Asendorpf et al., 2011; Cooper et al., 2012). In short, interpersonal romantic attraction is not driven by consensus but by individual preference (Kenny, 1988; Cooper et al., 2012).

In line with individual preference, the stimuli resulting in IRA actually belong to the category of stimuli causing appetite reaction based on individual preferences (Fisher, 1998; Fisher et al., 2005; Asendorpf et al., 2011; Zhang et al., 2011; Gerlach and Reinhard, 2018). Although there is a lack of research on the brain mechanism of IRA, there is an abundance of research on other appetitive responses elicited based on individual preference (Werheid et al., 2007; Schacht et al., 2008; Marzi and Viggiano, 2010; Van Hooff et al., 2011; Thiruchselvam et al., 2016; Ma et al., 2017). Neuroimaging studies have found that passive viewing of photograph content that elicits intense desire based on individual preference results in increased activation of emotion-processing and attention-control–related structures (Maas et al., 1998; Garavan et al., 2000; Itzhak Aharon et al., 2001; Quist and Chapela, 2002; Kranz and Ishai, 2006), such as posterior cingulate cortex (PCC), where (bottom-up) emotional information is drawn (Leech and Sharp, 2014; Spreng and Andrews-Hanna, 2015); orbitofrontal cortex (OFC), where emotional and motivational values are learned (O’Doherty et al., 2003; Dalgleish, 2004; Kranz and Ishai, 2006); and anterior cingulate cortex (ACC), where (top-down) attention is controlled (O’Doherty et al., 2003; Dalgleish, 2004; Kranz and Ishai, 2006). Interestingly, when participants were asked to make a social evaluation of the photograph content rather than just view it passively, neuronal activity in the dorsolateral prefrontal cortex (DLPFC) also increased significantly (Kranz and Ishai, 2006; Winston et al., 2007; Chatterjee et al., 2009; Ferrari et al., 2015). The DLPFC is thought to support social evaluation along several distinct dimensions, from PA to emotional valence (Katsuki et al., 1998; Ueda et al., 2003; Grimm et al., 2006, 2008; Winston et al., 2007; Chatterjee et al., 2009).

In parallel, several event-related potential (ERP) studies have reported enhanced late positive potential (LPP) amplitudes in response to photographs of cocaine among cocaine addicts (Herrmann et al., 2001; Franken et al., 2003, 2008; Dunning et al., 2011), and participants who were passionately in love also demonstrate increased LPP in response to photographs of their beloved (Langeslag et al., 2007, 2008). Meanwhile, some other studies have reported increased P300 amplitude in alcohol addicts when they see alcohol-related cues (Herrmann et al., 2001), as well as an increase in P300 amplitude when parents see their own infants (Weisman et al., 2012). Importantly, Hajcak et al. (2009) found that, compared with neutral stimuli, multiple increased late positivities, including LPP and P300, could be observed in the ERP after the presentation of emotional stimuli (Foti et al., 2009; Hajcak et al., 2010). Furthermore, in their comprehensive overview of ERPs, emotion, and emotion regulation, Hajcak et al. (2010) further indicated that the P300 may reflect automatic processing of emotional stimuli or a phasic increase in attention to intrinsic motivational stimuli, whereas the LPP is interpreted to signify controlled processing of emotional stimuli or a sustained increase in attention to intrinsically motivating stimuli (Foti et al., 2009; Hajcak et al., 2010).

**FIGURE 1:**
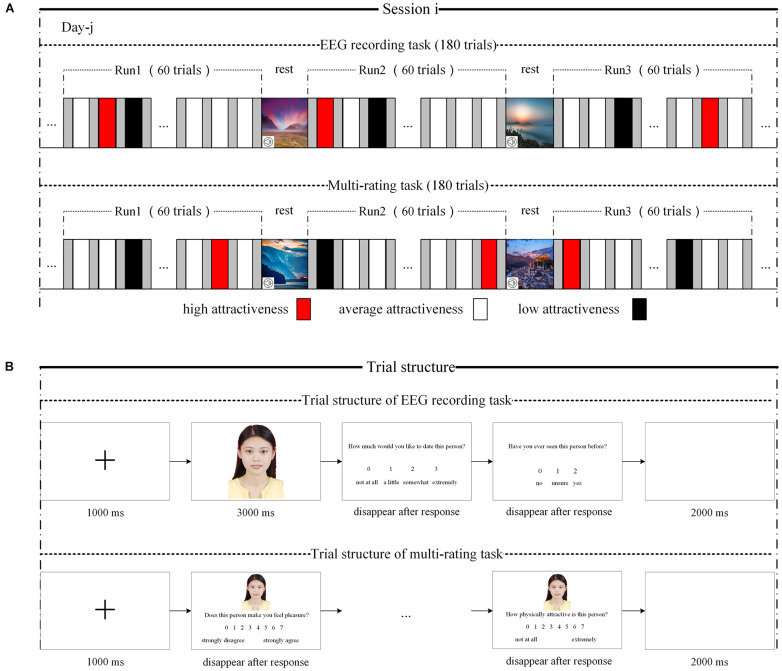
Experimental paradigm and trial structure. **(A)** Experimental paradigm. The upper part is the paradigm of EEG recording task, and the bottom part is the paradigm of multirating task. **(B)** Trial structure. The upper part is the trial structure of EEG recording, and the bottom part is the trial structure of multirating recording.

While the evolutionary value and dominant factors of IRA have been well established, little is known about the brain responses related to its generation and evaluation (Grant-Jacob, 2016; Zsok et al., 2017). To investigate the systems associated with generating and evaluating IRA, we used a simulated “online dating” paradigm (Figure 1A; Finkel et al., 2007; Cooper et al., 2012). Participants were asked to imagine dating the person in the photographs, and their electroencephalogram (EEG) signals were recorded in real time while they viewed photographs of each potential partner. The goal of the study was to identify the brain activities associated with the generation and evaluation of IRA.

First, during the EEG recording task, we asked heterosexual participants to rate photographs of opposite-sex potential partner on two dimensions: an “IRA” rating with the scale “How much would you like to date this person?” as well as a “zero acquaintance” rating with “Have you ever seen the person in the photograph before?” The scale (i.e., “How much would you like to date this person?”) was used to assess the IRA on the basis that (in humans) craving for emotional union with a potential partner is one of the main characteristics of IRA (Fisher, 1998; Fisher et al., 2002). Therefore, if an individual makes such a choice in the context of speed dating or online dating such as wanting to date this potential partner again, it can be taken as a sign of IRA (Finkel et al., 2007; Cooper et al., 2012; Gerlach and Reinhard, 2018). Following the EEG recording task, the participants were asked to perform a separate multirating task using the same set of photographs arranged in random order. On each self-paced trial, participants rated that photograph on a series of characteristics, including arousal, valence, dominance, and PA.

As with photographs of beloved people, cocaine (for addicts), one’s own baby, and alcohol (for addicts), faces that result in IRA belong to the category of stimuli that cause strong appetite response based on individual preference and thus belonged to the emotionally significant stimuli. Therefore, we hypothesized that the faces resulting in IRA could not only elicit larger LPP but also larger P300 and that these two larger late positivities would be mediated by distinct functional neural structures associated with automatic (such as PCC) and controlled processing (such as OFC and ACC) of emotional stimuli, respectively.

## Materials and Methods

Both the auxiliary experiment and the main experiment were approved by the Ethical Review Committee of Southwest University.

### Auxiliary Experiment

#### Participants

Participants were 60 student volunteers from Southwest University, who received payment for participating in the experiment. Participants were equally divided between males and females and ranged in age from 18 to 24 years (mean = 21.4, SD = 2.6). All participants reported normal or corrected to normal visual acuity and had no history of psychiatric or neurological disorders, as confirmed by a screening interview. All of them provided written informed consent.

#### Stimuli Task and Design

To investigate the systems associated with generating and evaluating IRA, we needed to first induce enough IRA reports (i.e., approximately 30 per participant) in the laboratory environment. However, the natural incidence of IRA is fairly low, approximately 1–2% (Zsok et al., 2017; Kerr et al., 2020). To achieve the goal of 30 IRA reports per participant, each participant would need to be exposed to at least 3,000 stimuli, a seemingly impossible task. Therefore, improving the induction rate of IRA was a challenge. Many studies have demonstrated that the consensus judgments of a potential partner’s PA is a good predictor of popularity (the probability of being chosen by the opposite sex) of both men and women (Kurzban and Weeden, 2005; Cooper et al., 2012; Zsok et al., 2017; Kerr et al., 2020). In particular, Zsok et al. (2017) found that a one-unit increase in PA level led to a several-fold increase in the induction rate of IRA. Thus, increasing the proportion of high-PA stimuli might increase the mean induction rate of IRA.

To increase the proportion of highly attractive stimuli, we first paid to download thousands of high-resolution personal portrait photographs from the high-definition copyright business photograph gallery website (such as VEER; Hummingbird net: http://bbs.fengniao.com/) and normalized these portrait photographs (face and hair only; size, 839 × 1,080 pixels). Second, to minimize confounds, 1,600 photographs (800 female faces) were chosen from the normalized portrait photographs, which were similar overall in face orientation and expression and comparable in background complexity; third, the PA of the 1,600 faces was rated using a nine-point rating scale (1 = “not at all” to 9 = “extremely”) in response to the question: “How physically attractive is this person?” with male participants rating only female faces and female participants rating only male faces. Finally, the consensus judgment of a face’s PA was calculated by averaging the attractiveness ratings of the same face over all participants.

### Main Experiment

#### Participants

This study included 130 college students (all single, 65 men, aged 18–24 years) attending Southwest University. All participants reported normal or corrected-to-normal visual acuity and had no history of psychiatric or neurological disorders, as confirmed by a screening interview. All of them provided written informed consent.

#### Stimuli Task and Design

In the natural environment, the proportion of faces with high, average, and low attractiveness should follow a normal distribution. In the present study, however, to increase the mean induction rate of IRA, we increased the proportion of highly attractive faces. According to the consensus judgment of PA, 360 photographs from the 800 photographs of women and from the 800 photographs of men were selected as the stimulus material of the main experiment. The 360 photographs of men were divided into three categories: high-attractiveness stimuli (mean = 6.7, SD = 0.36), average-attractiveness stimuli (mean = 5.1, SD = 0.29), and low-attractiveness stimuli (mean = 3.6, SD = 0.33). The 360 photographs of women were also divided into three categories: high-attractiveness stimuli (mean = 6.9, SD = 0.33), average-attractiveness stimuli (mean = 5.2, SD = 0.25), and low-attractiveness stimuli (mean = 3.9, SD = 0.31). Regardless of gender, the proportions of high-, average-, and low-attractiveness stimuli were approximately 0.25, 0.60, and 0.15, respectively.

Although the number of stimuli used in the main experiment was drastically reduced, 360 was still a large number of stimuli for participants. If participants were required to view 360 stimuli continuously over a short period, they may experience esthetic fatigue, which may then influence the experimental effect. To reduce the probability of esthetic fatigue, the 360 photographs of women (or men) were evenly distributed across two sessions according to attractiveness level; that is, 180 photographs were included in each session, and the proportions of high-, average-, and low-attractiveness photographs in each session were approximately 0.25, 0.6, and 0.15, respectively. The participants waited at least 24 h between sessions 1 and 2; furthermore, the 180 photographs in each session were evenly distributed into three runs according to attractiveness rating scores; that is, 60 photographs were included in each session, and the proportions of high-, average-, and low-attractiveness photographs in each session were about 0.25, 0.6, and 0.15, respectively. The stimuli in each run were presented in random order. A break was provided between every two runs during which participants viewed serene landscapes while listening to soothing music (5–6 min). It is worth noting that, to allow the participants to focus on the stimuli, the experiment was conducted in a dark and quiet environment.

For each session, participants first performed an EEG recording task (see upper part of Figure 1A). The trial was structured as follows (see upper part of Figure 1B). First, a black fixation cross appeared in the center of a white computer screen for 1,000 ms, followed by a face for 3,000 ms. The participants were then asked to rate their response to the question: “How much would you like to date this person?” using a four-point rating scale (“not at all,” “a little,” “somewhat,” or “very much”). Then, they were asked, “Have you ever seen the person in the photograph before?” (“no,” “not sure,” or “yes”). Finally, there was a 2,000-ms blank screen. The scale (i.e., “How much would you like to date this person?”) was used to assess the IRA on the basis that (in humans) craving for emotional union with a potential partner is one of the main characteristics of IRA (Fisher, 1998; Fisher et al., 2002). Therefore, if an individual makes such a choice in the context of speed dating or online dating such as wanting to date this potential partner again, it can be taken as a sign of IRA (Finkel et al., 2007; Cooper et al., 2012; Gerlach and Reinhard, 2018).

Following the EEG recording task in each session (and after removing the EEG recording device), the participants performed a separate multirating task (see bottom part of Figure 1A) using the same set of photographs arranged in the random order. On each self-paced trial (see bottom part of Figure 1B), the participants rated the photographs on a series of characteristics with seven-point scales, including four ratings of potential romantic desirability: “Does this person make you feel pleasure?” “Does this person make you feel excited?” “Does this person make you feel controlled?” and “How physically attractive is this person?”

#### Data Acquisition and Analysis

##### Electroencephalogram recording

The EEG analog signals were recorded using a 128-channel BioSemi ActiveTwo system (BioSemi Inc., Netherlands) with 24-bit analog-to-digital conversion. The 128 electrodes were equally spaced on an electrode cap and customized with an integrated primary amplifier. Data were filtered online with a 0.16- to 100-Hz bandpass filter and sampled at 512 Hz (Muller et al., 2014).

##### Data processing

The EEG data were re-referenced offline to the average of all channels after rejecting bad segments and interpolating bad traces; the bandpass filtered between 0.1 and 40 Hz (12 dB/octave) and corrected for eye movements using the algorithm of Gratton et al. (1983). An independent component analysis procedure was used to correct the EEG deflections resulting from eye movements and blinks (Jung et al., 2000; Perry et al., 2013). Remaining artifact rejection criteria were minimum and maximum baseline-to-peak 75 to +75 mV with a maximum allowed voltage skip (gradient) of 50 mV (Langeslag et al., 2007). Data were split into epochs from 100 ms before presentation of the stimulus to 2,000 ms after stimulus onset.

These EEG epochs were categorized according to rating score (0 = “not at all” to 3 = “very much”) based on the question: “How much would you like to date this person?” (Cooper et al., 2012; Zsok et al., 2017). Epochs with a rating score of 2 or 3 were assigned to the IRA-engendered condition, and epochs with a rating score of 0 were assigned to the IRA-unengendered condition. To minimize ambiguity, epochs with a rating score of 1 were excluded (mean = 19.78, SD = 3.12). It is worth noting that one additional type of epochs was excluded: epochs corresponding to faces the participants had seen or were not sure they had seen were excluded (mean = 1.93, SD = 2.54). The mean number of accepted epochs per participant under the IRA-engendered condition was 34.58 (SD = 3.38), and the mean number of accepted epochs per participant under the IRA-unengendered condition was 303.71 (SD = 6.31). To solve the serious mismatch between the two conditions of the number of trials, for each participant, a number of accepted epochs under the IRA-unengendered condition were randomly selected to match the number of accepted epochs under IRA-engendered condition.

### Behavioral and Electroencephalogram Analysis

#### Behavioral Analysis

We first examined the extent to which the IRA measure was related to the consensus ratings of PA with Pearson correlation coefficient, as well as calculated the mean elicitation rate of IRA among high-, average-, and low-PA stimuli. Furthermore, Pearson correlation coefficient was also used to examine the relationship between the IRA scores and each of the four characteristics (i.e., arousal, valence, dominance, and PA) scores.

Pearson correlation coefficient is the most common measure to evaluate the linear correlation between two sets of data. It is the ratio between the covariance of two variables and the product of their standard deviations.


(1) $$r\,\left(X,Y\right)=\frac{C\,o\,v\,\left(X,Y\right)}{\sigma_{X}\,\sigma_{Y}}=\frac{\sum_{i=1}^{n}\left(X_{i}-\bar{X}\right)\,\left(Y_{i}-\bar{Y}\right)}{\sqrt{\sum_{i=1}^{n}{\left(X_{i}-\bar{X}\right)}^{2}}\,\sqrt{\sum_{i=1}^{n}{\left(Y_{i}-\bar{Y}\right)}^{2}}}$$

where *X* and *Y* represent the two data sets used to calculate the correlation (e.g., PA score and IRA score). In essence, Pearson correlation coefficient is a normalized measurement of the covariance, such that the coefficient always has a value between −1 and 1. The absolute value of Pearson correlation coefficient reflects the extent of correlation. The closer the absolute value of the coefficient is to 1, the higher the correlation between the two variables. The closer the absolute value of the coefficient is to 0, the lower the correlation between the two variables. The sign of Pearson correlation coefficient determines the direction of correlation. A positive sign indicates the phase of the change trend of the two variables. A negative sign indicates that the change trend of the two variables is opposite.

#### Electroencephalogram Analysis

To test whether the faces that resulted in IRA induce larger P300 and larger LPP, and whether these two larger late positivities would be mediated by distinct functional neural structures, we performed an ERP analysis and further dipole-source analysis.

**FIGURE 2:**
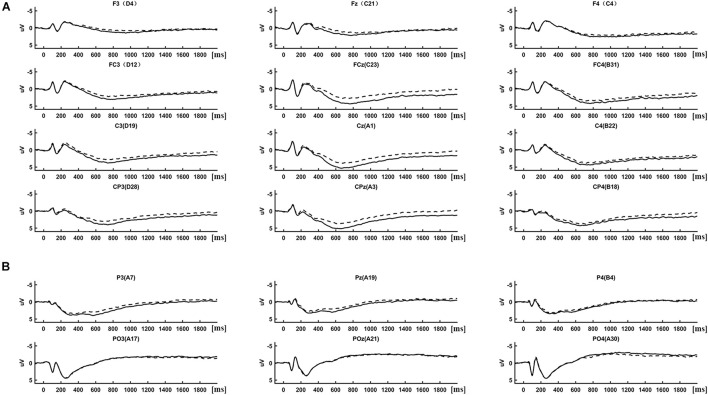
ERP differences between IRA engendered and IRA un-engendered condition, both at the LPP **(A)** and the P300 **(B)**.

**FIGURE 3:**
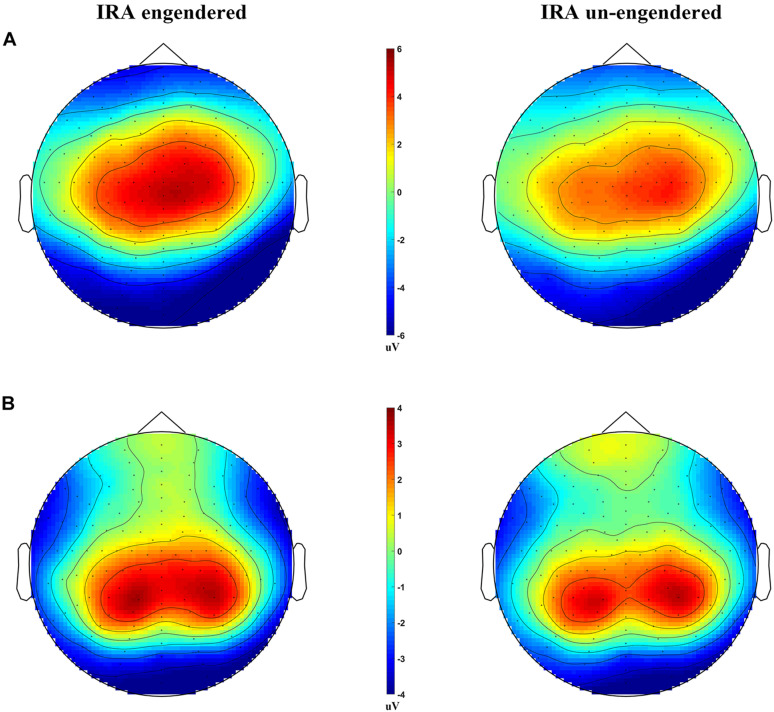
Scalp distributions of the LPP and P300, for the IRA engendered and IRA un-engendered conditions. **(A)** The LPP. **(B)** The P300.

(1) Event-related potential analysis: ERPs were determined by averaging the 2-s segmented trials separately in each condition (IRA-engendered, IRA-unengendered). The results of the ERP analysis are presented in Figures 2, 3. As there were obvious differences among the latencies of late positivities between anterior regions (F3/4, Fz, FC3/4, FCz, C3/4, Cz, CP3/4, and CPz; Figure 2A) and occipital–parietal regions (P3/4, Pz, PO3/4, and POz; Figure 2B), we analyzed the peak latency of late positivities before conducting a statistical analysis of the peak amplitude. The peak latencies of late positivities were tested using a repeated-measures analysis of variance (ANOVA) design for the within-subject factor Location (two levels: anterior regions and occipital–parietal regions) and Condition (two levels: RAZA engendered, RAZA unengendered). The results of the repeated-measures ANOVA showed that the interaction effect between Location and Condition was not significant (*F*_1,125_ = 0.47, *p* > 0.05). The main effect analysis of simple factors showed there was a significant main effect for Location over the parietal regions (*F*_1,125_ = 8.62, *p* < 0.05), revealing that the mean peak latencies of late positivities over the anterior regions (767.42 ms, SD = 6.32 ms) were significantly longer than those over the occipital–parietal regions (289.67 ms, SD = 14.36 ms). There were no other significant main effects or interactions. These findings suggest the late positivities over the occipital–parietal regions is P300, whereas the positivities over the anterior regions is LPP (Foti et al., 2009; Hajcak et al., 2010).

Based on previous studies and on scrutiny of the present distributions (Figures 2, 3), the statistical analysis of the P300 components was restricted to the occipital–parietal sites P3/4, Pz, PO3/4, and POz, and the statistical analysis of the LPP components was restricted to the parietal–central–frontal sites F3/4, Fz, FC3/4, FCz, C3/4, Cz, CP3/4, and CPz (Foti et al., 2009; Hajcak et al., 2010; Marzi and Viggiano, 2010; Zhang and Deng, 2012; Ma et al., 2017). For each subject, the peak of P300 component was determined as the most positive peak between 250 and 400 ms (Lehmann and Skrandies, 1980; Pizzagalli et al., 2002; Foti et al., 2009; Hajcak et al., 2010), and the peak of LPP was determined as the most positive peak between 600 and 1,000 ms (Foti et al., 2009; Hajcak et al., 2010). Subsequent visual scrutiny ensured that these values represented real peaks rather than end points of the epoch. The statistical analysis of each peak was based on a repeated-measures ANOVA design (Location, Condition) for the amplitude of each peak.

(2) Standardized low-resolution brain electromagnetic tomography (SLORETA) analysis: First, the difference between the average IRA-engendered response and the average IRA-unengendered response was calculated at each time frames of the 128 channels using the paired-sample *t* test (Ghiselli et al., 2020). Multiple-comparison corrections based on randomization statistical non-parametric mapping (5,000 times) were used to assess the statistical significance of all *t* values (*p* < 0.05). The paired-sample *t* test results show significant differences in two time ranges: one from 298 to 396 ms (the common range of P300 latency) (Foti et al., 2009; Hajcak et al., 2010), and the other from 448 to 932 ms (the common range of LPP latency) (Foti et al., 2009; Hajcak et al., 2010). Then, at the individually determined latency stages of P300 and LPP, the cortical three-dimensional distribution of current source density (CSD) for IRA-engendered and IRA-unengendered condition was computed using SLORETA (Fuchs et al., 2002; Pascual-Marqui, 2002; Jurcak et al., 2007). For this purpose, the time-domain scalp activity recorded at each electrode was first converted into the current CSD field on a three-dimensional source space (6,239 cerebrospinal gray matter voxels with a resolution of 5 mm) based on the transformation matrix (Csukly et al., 2013; Sasaki et al., 2019). Then, voxel-by-voxel one-sample *t* tests on log-transformed data between the two conditions were used to determine whether these two larger late positivities would be mediated by distinct functional neural structures. Multiple-comparison corrections based on randomization statistical non-parametric mapping (5,000 times) were used to assess the statistical significance of all *t* values (*p* < 0.05).

## Results

### Behavioral Data

A positive correlation was found between the IRA scores and the consensus ratings of PA (*R* = 0.32, *p* < 0.01), such that the higher a potential partner’s PA level, the greater the likelihood that the potential partner would be of romantic interest to the participants. Specifically, the average induction rates of IRA for faces with low attractiveness, average attractiveness, and high attractiveness were 0.00, 0.06, and 0.25, respectively.

In addition, when arousal, valence, dominance, and PA measures were included separately in a correlated model, attractiveness was significantly related to all four measures (IRA-arousal: *R* = 0.75, *p* < 0.01; IRA-valence: *R* = 0.67, *p* < 0.01; IRA-dominance: *R* = 0.73, *p* < 0.01; IRA-PA: *R* = 0.71, *p* < 0.01). Furthermore, because the four measures of arousal, valence, dominance, and PA were also highly correlated with each other (*R*’s > 0.61, *p*’s < 0.01), partial correlation models were used to examine the unique contribution of each measure. Partial correlations revealed that the measures of arousal and dominance ratings accounted for the majority of the variance underpinning IRA ratings (mean within-participant IRA-arousal partial correlation = 0.29, *p* < 0.001; mean within-participant IRA-valence partial correlation = 0.21, *p* < 0.001; mean within-participant IRA-dominance partial correlation = 0.23, *p* < 0.001; mean within-participant IRA-PA partial correlation = 0.14, *p* < 0.001). These analyses therefore indicated that increasing the proportion of high attractiveness faces indeed significantly increased the mean IRA induction rates, suggesting indirectly that consensus judgment of PA was a significant predictor of a partner’s popularity (Finkel et al., 2007; Asendorpf et al., 2011). Furthermore, a participant’s romantic interest in a potential partner was most associated with individual judgments of arousal and dominance, which is consistent with the viewpoint that IRA in humans is often accompanied by feelings of excitement and intrusive thinking about potential partners (Fisher, 1998; Fisher et al., 2002; Cooper et al., 2012).

### Electroencephalogram Data

The distribution of the LPP was parietal–central–frontal (Figure 3A; Foti et al., 2009; Hajcak et al., 2010), and the distribution of the P300 was occipital–parietal (Figure 3B; Foti et al., 2009; Hajcak et al., 2010). The mean peak latency of the P300 was 289.67 ms (SD = 14.36 ms), and the mean peak latency of the LPP was 767.42 ms (SD = 6.32 ms). The statistical analysis of each peak was based on a repeated-measures ANOVA design (Location, Condition) for the amplitude of each peak. The ERP data were from 125 participants (61 male and 64 female participants) because of incomplete behavioral data for three participants and poor quality of EEG data for the other two subjects (frequent and large head movements).

#### Late Positive Potential

There was a significant interaction effect between Location and Condition (*F*’s_1,125_ = 2.95, *p* < 0.05). The main effect analysis of simple factors showed that the main effect of Condition was significant at all levels of Location (*F*’s_1,125_ = 15.16, *p*’s < 0.001). *Post hoc* comparisons showed that, compared with stimuli that did not result in IRA, stimuli that resulted in IRA elicited larger LPP over frontal (IRA-engendered = 1.76, IRA-unengendered = 1.20; Bonferroni-correction *p* < 0.001; Figure 2A), frontocentral (IRA-engendered = 3.61, IRA-unengendered = 2.61; Bonferroni-correction *p* < 0.001), central (IRA-engendered = 4.22, IRA-unengendered = 3.27; Bonferroni-correction *p* < 0.001), and centroparietal regions (IRA-engendered = 4.07, IRA-unengendered = 3.14; Bonferroni-correction *p* < 0.001). In addition, the main effect of Location was significant at all levels of the Condition (*F*’s_1,125_ = 75.69, *p*’s < 0.001). *Post hoc* comparisons showed that both in the condition of IRA-engendered and in the condition of IRA-unengendered (1) there was no significant difference of LPP over central (IRA-engendered/unengendered: 4.22/3.27) and centroparietal regions (IRA-engendered/unengendered: 4.07/3.13; Bonferroni-correction *p* > 0.05); (2) the LPP over central and centroparietal regions were both significantly larger than that over the frontocentral region ((IRA-engendered/unengendered: 3.61/2.60; Bonferroni-correction *p*’s < 0.05), whereas the LPP over the frontocentral region was significantly larger than that over the frontal region (IRA-engendered/unengendered: 1.76/1.20; Bonferroni-correction *p*’s < 0.001). There were no other significant main effects or interactions.

#### P300

There was a significant interaction effect between Location and Condition (*F*_1,125_ = 5.22, *p* < 0.05). The main effect analysis of simple factors showed that there was a significant main effect for Condition over the parietal regions (*F*_1,125_ = 3.45, *p* < 0.05), revealing that P300 was greater for the IRA-engendered (2.86) than for the IRA-unengendered condition (2.31; Figure 2B). There were no other significant main effects or interactions.

**FIGURE 4:**
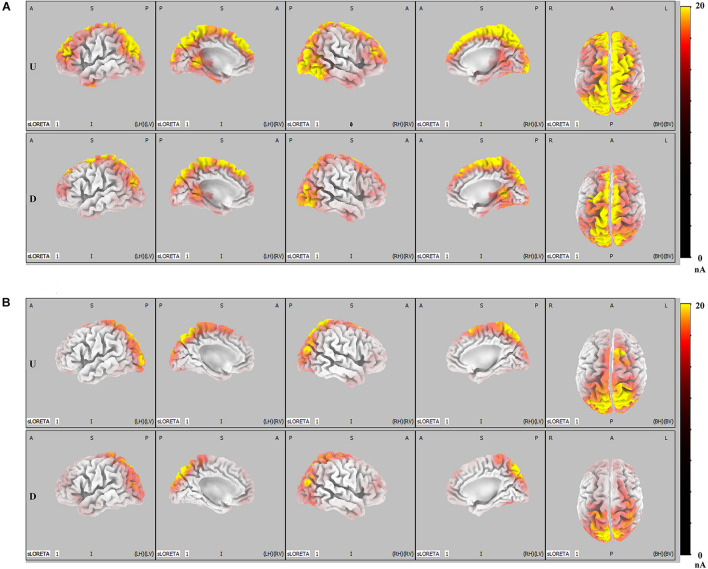
LORETA probabilistic map in cortical activation of LPP and P300, for the IRA engendered and IRA un-engendered conditions. Red colors represent a greater activation, more evident from dark red to yellow. **(A)** The LPP. **(B)** The P300.

Results of ERP analysis demonstrated that, when compared with faces that did not result in IRA, faces that resulted in IRA elicited a larger P300 over the parietal region and enhanced LPP over the parietal–central–frontal regions. To further determine whether these two larger late positivities would be mediated by distinct functional neural generators, we performed further SLORETA analysis. Both in the condition of romantic attraction and in the condition of non-romantic attraction, the estimated P300 sources were mainly localized in the parietal–occipital regions and partial emotional processing areas (*p* < 0.01, Figure 4B); however, the estimated LPP sources were not only in the parietal–occipital regions and partial emotional processing areas, but also extended forward to the prefrontal regions (*p* < 0.01, Figure 4A).

**FIGURE 5:**
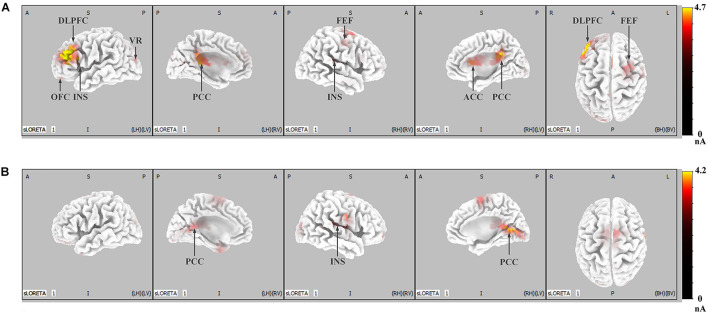
LORETA probabilistic map in cortical difference of activation between IRA engendered and IRA un-engendered conditions, both at the LPP **(A)** and the P300 **(B)**. Red colors represent a greater activation in IRA engendered condition, more evident from dark red to yellow.

Figure 5 shows the difference in cortical activation within each time window. At the LPP latency stage (448- to 932-ms time window; Figure 5A), there was greater activation in the ACC, OFC, frontal eye field (FEF), visual regions (VRs), insula, PCC, and DLPFC in IRA-engendered condition (*p* < 0.05). At the P300 latency stage (298- to 396-ms time window; Figure 5B), neuron activity in the insula and PCC significantly increased when IRA was engendered compared to activation in those regions in the absence of IRA (*p* < 0.05). These findings suggest that larger P300 response magnitude over the parietal lobe may be caused by stronger activation of the insula and PCC, whereas enhanced LPP over the prefrontal and central cortices may be caused by heightened activity in the ACC, OFC, DLPFC, FEF, VAC, insula, and PCC neurons.

Generally, these results indicate that, compared with faces that did not result in IRA, faces that resulted in IRA evoked a larger P300 response magnitude over the parietal regions and heightened neuron activity in the insula and PCC; they also elicited enhanced LPP over the parietal–central–frontal regions and stronger neural activation in the OFC, ACC, FEF, VR, insula, PCC, and DLPFC.

## Discussion

Traditionally, generating IRA is the first step in any romantic relationship, and it largely determines whether an individual has the motivation or desire to interact further with potential mates, thereby helping individuals rationally allocate limited mating resources and time. The aim of the present study was to investigate the neural activity associated with the generation and evaluation of IRA by combining classic ERP analysis with dipole-source analysis. Several findings relevant to the initial hypotheses emerged:

On the one hand, compared with the faces that did not result in IRA, faces that result in IRA elicited larger P300. This finding is in line with Weisman and Herrmann, who found stimuli that elicit intense desire based on individual preference elicit relatively large P300 (Herrmann et al., 2001; Weisman et al., 2012). On the other hand, faces that result in IRA elicited larger LPP. This finding is in line with Langeslag and Franken, who reported that photographs of preferred objects appear to elicit an increased LPP (Langeslag et al., 2007; Franken et al., 2008; Dunning et al., 2011). Our study thus provides additional support for the viewpoint that individual differences in preference for stimuli relate to variation in the late positivity (e.g., P300 or LPP). More importantly, to the best of our knowledge, our study demonstrates for the first time that the faces that result in IRA simultaneously elicit multiple relatively large late positivities, namely, P300 and LPP. These two different late positivities not only had different peak latency and different distribution patterns, but also are modulated by different brain regions: the mean peak latency of P300 was 289.67 ms, which was most significant in the occipital–parietal region and was mainly modulated by the activity of the insula and PCC, whereas the average peak latency of LPP was 767.42 ms, which was most significant in a wide area from the parietal region to the frontal lobe and was mainly modulated by the activity of insula, cingulate cortex, and OFC, DLPFC, and FEF. It follows that these two distinct different late positivities may be involved in different cognitive processes during IRA.

On the one hand, many early ERP studies found that, compared with neutral stimuli, emotional stimuli induced a larger P300 response (Mini et al., 1996; Foti et al., 2009; Hajcak et al., 2010), which is considered to reflect automatic processing of emotional stimuli or a phasic increase in attention to intrinsic motivational stimuli (Foti et al., 2009; Hajcak et al., 2010). Interestingly, functional magnetic resonance imaging (fMRI) studies consistently show that the PCC, which has extensive connections with the limbic and paralimbic regions, plays an important role in supporting bottom-up attention to emotional information from memory and/or perception (Leech and Sharp, 2014; Spreng and Andrews-Hanna, 2015). Furthermore, as a key component of the limbic system, the insula plays an important roles in emotional stimuli processing (Davidson and Irwin, 1999; Phan et al., 2002; Morgane et al., 2005; Grimm et al., 2006). Specifically, the insula is preferentially involved in the evaluation of internal emotions and the formation of subjective feelings such as maternal and romantic love, happiness, sadness, and sexual arousal (Phan et al., 2002; Grimm et al., 2006; Craig, 2009). As the larger P300 response in the parietal lobe was caused by enhanced neural activation in the insula and PCC, these evidences of convergence may indicate that P300 may reflect involvement of the limbic system in the extraction of basic emotional information from stimuli and forming preliminary subjective feelings. Limbic system involvement can regulate the attention system, which allows for an increase in phasic attention to intrinsic motivational stimuli.

On the other hand, recent ERP studies have found that, when compared with neutral stimuli, stimuli that cause appetitive response based on individual preference evoke a larger LPP (Kolassa et al., 2005; Langeslag et al., 2007; Marzi and Viggiano, 2010; Zhang and Deng, 2012); this is interpreted to signify controlled processing of emotional stimuli or a sustained increase in attention to intrinsically motivating stimuli (Hajcak et al., 2010; Marzi and Viggiano, 2010; Van Hooff et al., 2011; Thiruchselvam et al., 2016; Ma et al., 2017). Like the PCC, the FEF plays an important role in attention control (Vossel et al., 2014; Ptak et al., 2017). However, unlike the PCC, which supports bottom-up attention capture (Leech and Sharp, 2014; Spreng and Andrews-Hanna, 2015), the FEF, as a component of the dorsal attention system, aids in top-down attention allocation driven by motivational significance (Corbetta and Shulman, 2002; Vossel et al., 2014; Ptak et al., 2017; Lowe and Schall, 2018). Like the insula, ACC and OFC are also key components of the limbic system (Phan et al., 2002; Dalgleish, 2004). The ACC, as the key integration point of visceral, attention, and emotional information, plays a crucial role in the recognition of emotion and other forms of top-down control (Davidson et al., 2002; Phan et al., 2002; Grimm et al., 2006), whereas the OFC is involved in learning the emotional and motivational value of stimuli (O’Doherty et al., 2003; Dalgleish, 2004; Kranz and Ishai, 2006). As enhanced LPP in the prefrontal and central cortices is caused by heightened neuronal activity in the ACC, OFC, and FEF, it follows that enhanced LPP may reflect engagement of the limbic systems to promote the formation of reward motivation that can drive the dorsal attention system and involvement of the top-down attention system to facilitate perception.

In addition to ACC, OFC, and FEF, LPP is also modulated by the DLPFC (the main area of neuronal activity enhancement in the present study). fMRI studies demonstrate that the DLPFC plays an important role in decision-making processes (Grimm et al., 2006; Chatterjee et al., 2009), such as emotion–valence evaluation (Davidson and Irwin, 1999; Ueda et al., 2003; Northoff et al., 2004; Grimm et al., 2006) and face–attractiveness judgment (Kranz and Ishai, 2006; Chatterjee et al., 2009; Ferrari et al., 2015). In particular, many studies have found that activation of the left DLPFC is significantly increased when participants are exposed to stimuli that induce positive emotions (Davidson and Irwin, 1999; Boggio et al., 2007; Grimm et al., 2008; Balconi and Ferrari, 2013). Therefore, we propose that stronger DLPFC activity may also reflect the involvement of higher-level cognitive control structures to evaluate whether faces result in romantic interest.

Taken together, these findings extend the previous findings that stimuli that cause intense appetite response based on individual preference elicit relatively large positivity (such as P300 or LPP), thus revealing that not only faces that resulted in IRA elicited larger P300 over the parietal region and larger LPP over the parietal–central–frontal regions, but also these two brain responses may represent different cognitive processes during IRA. Specifically, the larger P300 in the parietal lobe may reflect that limbic system participates in extracting basic emotional information from stimuli and then regulates the attention system, thus increasing the early phasic attention to intrinsic motivation stimuli, whereas the enhanced LPP may reflect that limbic system participates in the formation of reward motivation and then drives the dorsal attention system to allocate attention from top to bottom to promote perception.

Several limitations to this study should be acknowledged; first, we used the dipole-source method to study neural activities associated with the generation and evaluation of IRA. However, because of the inherent deficiency of EEG’s detection depth, we were unable to detect the neural activity of key emotion-processing structures (such as the basal ganglia) buried deep in the lower cortex. This means we were unable to grasp the whole photograph of the brain mechanisms involved in romantic attraction. Higher spatial resolution tools, such as fMRI, are needed in future research. In addition, although it is convenient and efficient to use portraits to evoke and study brain activities related to IRA, the intensity of emotional arousal is weaker than that in response to real people. Therefore, follow-up studies should use real people to arouse and study romantic attraction.

## Data Availability Statement

The raw data supporting the conclusions of this article will be made available by the authors, without undue reservation.

## Ethics Statement

The studies involving human participants were reviewed and approved by The Ethical Review Committee of Southwest University. The patients/participants provided their written informed consent to participate in this study.

## Author Contributions

All authors conceived the study and approved the final manuscript. GY designed and programmed the tasks, contributed to data collection, analyzed the composite behavioral data and EEG data, and wrote the manuscript. GY and DW interpreted the results. GL and DW provided revisions to the manuscript.

## Conflict of Interest

The authors declare that the research was conducted in the absence of any commercial or financial relationships that could be construed as a potential conflict of interest.

## Publisher’s Note

All claims expressed in this article are solely those of the authors and do not necessarily represent those of their affiliated organizations, or those of the publisher, the editors and the reviewers. Any product that may be evaluated in this article, or claim that may be made by its manufacturer, is not guaranteed or endorsed by the publisher.
